# The Association between Recent Cannabis Use and Suicidal Ideation in Adults: A Population-based Analysis of the NHANES from 2005 to 2018

**DOI:** 10.1177/0706743721996112

**Published:** 2021-03-01

**Authors:** Calvin Diep, Venkat Bhat, Duminda N. Wijeysundera, Hance A. Clarke, Karim S. Ladha

**Affiliations:** 1 Department of Anesthesiology and Pain Medicine, University of Toronto, Ontario, Canada; 2 Department of Psychiatry, University of Toronto, Ontario, Canada; 3 Department of Psychiatry, St. Michael’s Hospital, Toronto, Ontario, Canada; 4 Li Ka Shing Knowledge Institute, St. Michael’s Hospital, Toronto, Ontario, Canada; 5 Faculty of Medicine, Institute of Medical Science, University of Toronto, Ontario, Canada; 6 Institute of Health Policy, Management, and Evaluation, University of Toronto, Ontario, Canada; 7 Department of Anesthesia, St. Michael’s Hospital, Toronto, Ontario, Canada; 8 Department of Anesthesia and Pain Management, Toronto General Hospital, Ontario, Canada

**Keywords:** cannabis, suicidal ideation, depression, NHANES

## Abstract

**Objective:**

With the increasing prevalence of cannabis use, there is a growing concern about its association with depression and suicidality. The aim of this study was to examine the relationship between recent cannabis use and suicidal ideation using a nationally representative data set.

**Methods:**

A cross-sectional analysis of adults was undertaken using National Health and Nutrition Examination Survey data from 2005 to 2018. Participants were dichotomized by whether or not they had used cannabis in the past 30 days. The primary outcome was suicidal ideation, and secondary outcomes were depression and having recently seen a mental health professional. Multiple logistic regression was used to adjust for potential confounders, and survey sample weights were considered in the model.

**Results:**

Compared to those with no recent use (*n* = 18,599), recent users (*n* = 3,127) were more likely to have experienced suicidal ideation in the past 2 weeks (adjusted odds ratio [aOR] 1.54, 95% CI, 1.19 to 2.00, *P* = 0.001), be depressed (aOR 1.53, 95% CI, 1.29 to 1.82, *P* < 0.001), and to have seen a mental health professional in the past 12 months (aOR 1.28, 95% CI, 1.04 to 1.59, *P* = 0.023).

**Conclusions:**

Cannabis use in the past 30 days was associated with suicidal thinking and depression in adults. This relationship is likely multifactorial but highlights the need for specific guidelines and policies for the prescription of medical cannabis for psychiatric therapy. Future research should continue to characterize the health effects of cannabis use in the general population.

## Abstract (fr)

**Objectif:**

Avec la prévalence croissante de l’usage du cannabis, on se préoccupe de plus en plus de l’association de celui-ci avec la dépression et la suicidabilité. La présente étude avait pour but d’examiner la relation entre l’usage récent du cannabis et l’idéation suicidaire au moyen d’un ensemble de données nationalement représentatives.

**Méthodes:**

Une analyse transversale d’adultes a été entreprise à l’aide des données de l’Enquête nationale sur l’examen de la santé et de la nutrition (NHANES) de 2005 à 2018. Les participants ont été dichotomisés selon qu’ils avaient ou non utilisé du cannabis dans les 30 jours précédents. Le principal résultat était l’idéation suicidaire et les résultats secondaires étaient la dépression et le fait d’avoir récemment consulté un professionnel de la santé mentale. La régression logistique multiple a servi à ajuster des facteurs de confusion éventuels et les pondérations de l’échantillon de l’enquête ont été considérées dans le modèle.

**Résultats:**

Comparés à ceux sans utilisation récente (n = 18 599), les utilisateurs récents (n = 3 127) étaient plus susceptibles d’avoir eu une idéation suicidaire dans les deux semaines précédentes (RCa 1,54; IC à 95% 1,19 à 2,00: p = 0,001), d’être déprimés (RCa 1,53; IC à 95% 1,29 à 1,82; p < 0,001), et d’avoir consulté un professionnel de la santé mentale dans les 12 mois précédents (RCa 1,28; IC à 95% 1,04 à 1,59; p = 0,023).

**Conclusions:**

L’usage du cannabis dans les 30 jours précédents était associé à l’idéation suicidaire et à la dépression chez les adultes. La relation est probablement multifactorielle mais elle souligne le besoin de lignes directrices et de politiques spécifiques pour la prescription de cannabis à des fins médicales dans le cadre d’une thérapie psychiatrique. La recherche future devrait continuer de caractériser les effets sur la santé de l’usage du cannabis dans la population générale.

## Introduction

North America has the greatest number of cannabis users of any region in the world, and the prevalence continues to grow.^1^ This is attributed, at least in part, to widespread legalization and decriminalization in recent years alongside changes in attitude toward cannabis for its potential therapeutic properties.^2,3^ The 2019 National Cannabis Survey revealed that nearly half of all Canadians will use cannabis at least once in their lifetime, and 35% of young adults and 18% of the general population had done so in the past 3 months alone.^4^

Despite its perceived safety profile, cannabis use has been shown to be associated with reduced function and adverse outcomes related to cardiovascular and respiratory health,^5–7^ cognition,^8^ psychosis,^9^ and depression.^10–12^ A recent study using the U.S.-based, nationally representative data set from the National Health and Nutrition Examination Survey (NHANES) found that adults with depression had greater odds of cannabis use in the past month compared to those without depression.^13^ Individuals with depression who also use cannabis are postulated to be at higher risk for further adverse mental health outcomes.^14^ There are concerns about increased suicidal ideation or suicide attempts with acute or chronic cannabis use, though there is insufficient evidence to claim causality.^5,15^ Two meta-analyses^10,14^ have demonstrated an association between the 2, although the included studies do not reflect current use patterns and have nonrepresentative samples of the general population.

Given the immense and tragic burden that self-harm, suicidal ideation, and suicidal behavior have at the individual and population levels, their relationships to increasing cannabis use are important to investigate in order to identify contributing factors. Therefore, we sought to characterize the association between suicidal ideation and recent cannabis use in a nationally representative data set.

## Methods

### Study Population

Data for this study were obtained from the NHANES. This is a cross-sectional survey administered by the National Center for Health Statistics (NCHS) and Centers for Disease Control and Prevention. The NHANES is designed to yield nationally representative data for the noninstitutionalized civilian population in the United States. This is achieved using a multistage area probability sample selection: (1) selection of primary sampling units (PSUs), (2) segments within PSUs (1 or more blocks containing a cluster of households), (3) households within segments, and (4) at least 1 participant within each household. Sample weights and adjustments are then made to account for oversampling and control for nonresponse.^16–19^ The data collection protocols are approved by the NCHS Ethics Review Board, and all survey participants provide informed consent prior to being interviewed and examined. For this present study, a data set was constructed using publicly available data files with NHANES responses from 2005 to 2018. The study population consisted of all respondents to NHANES cannabis questionnaires, which were only administered to those aged 20 to 59 years.

### Exposure

Cannabis use was the primary exposure variable for this study. Participants were categorized as “recent” users if they had used cannabis in the past 30 days or “no recent use” if they had never used or had not used in the past 30 days. In a secondary analysis, recent cannabis users were characterized as “moderate” or “heavy” users if they had respectively used for <20 or ≥20 days in the past 30 days. While there is no consensus on the definition of heavy cannabis use, this threshold was used to remain consistent with previous studies.^13,20^

### Outcomes

The primary outcome for this study was suicidal ideation, as defined by the response to Item #9 of the Patient Health Questionnaire-9 (PHQ-9) administered as part of the NHANES. The PHQ-9 is a validated screening instrument composed of 9 items for identifying the presence and severity of symptoms of clinical depression in the past 2 weeks.^21^ Each item is scored from 0 to 3, with 0 indicating that the symptom was experienced on no days in the past 2 weeks, 1 if on several days, 2 if on more than half the days, and 3 on nearly every day. Item #9 specifically asks respondents whether they have experienced thoughts of being dead or thoughts of hurting themselves in some way in the past 2 weeks. Suicidal ideation reported by Item #9 has been shown to be a robust predictor of attempted or completed suicide in the following weeks to months.^22,23^ The presence of clinical depression was considered as a secondary outcome. Depression status was dichotomized as a summed PHQ-9 score of either <10 (not/minimally or mildly depressed) or ≥10 (moderately or severely depressed). This cutoff value was chosen for its high sensitivity and specificity for major depression.^21^ An additional secondary outcome considered was whether or not the survey participant reported seeing a mental health professional such as a psychologist, psychiatrist, psychiatric nurse, or clinical social worker in the past 12 months.

### Covariates

Covariates were selected a priori based on biological plausibility of being a confounder in the relationship between the exposure and primary outcome.^13,24,25^ Demographic characteristics such as age (categorized as 20 to 29 years old, 30 to 39, 40 to 49, or 50 to 59), sex, and race (grouped as Hispanic, non-Hispanic white, non-Hispanic black, or other) were included. Level of education (dichotomized as high school diploma or below vs. any training above high school diploma), marital status (categorized as never married, married or living with partner, or separated/divorced/widowed), number of people living in the household (categorized as living alone, 2 to 4 people, or ≥5 people), ratio of family income to poverty level (categorized as ≤1, 1 to 3, or >3), and health insurance coverage were included as socioeconomic covariates. Data about health-related behaviors such as cigarette smoking (categorized as never [<100 cigarettes in life], former [>100 cigarettes but not currently smoking], or current [smoking “some days” or “every day”]), current alcohol use (none, moderate [<5 drinks per day and <15 per week for males, and <4 per day and <8 per week for females], or heavy [meeting or exceeding those thresholds]), and other drug use (having ever used cocaine, heroin, or methamphetamines) were collected. Variables related to medical comorbidities such as body mass index (BMI; categorized as <25 kg/m^2^, 25 to 30 kg/m^2^, or ≥30 kg/m^2^), diabetes, arthritis, and cancer were also included. Finally, the 2-year survey cycle during which the data were collected was included in the model.

### Missing Data

Survey questions related to the alcohol use covariate were not yet published for the 2017 to 2018 cycle at the time this study was conducted. To retain this large sample of participants in analyses, an additional level was coded into the alcohol use variable labeled “missing data.” Similarly, for any covariate for which >5% of survey participants were missing data, a separate level was coded for the missing values. Respondents with missing values for any other covariates were removed from the analyses.

### Data Analysis

Weighted differences in cohort characteristics and outcome variables across exposure groups were analyzed using χ^2^ tests. Multivariable logistic regression was used to determine the association between the exposure and outcome variables in the primary analysis while accounting for covariates. Regression models took into consideration survey sample weights accompanying the NHANES data. All covariates were included in the model without further selection. In a post hoc analysis, an interaction term between cannabis use and the year of survey administration was added to the regression model. The interaction term was found to be not statistically significant and thus was not included in the primary analysis. No other interaction terms were considered.

Additional subgroup analyses were performed—one stratifying by sex and another by age-group—to determine the impact of these subgroups on effect sizes in the relationship between our exposure and outcome variables. A secondary analysis was conducted by modifying the definition of the exposure variable to further characterize recent cannabis users as moderate (use on <20 days per month) or heavy (≥20 days per month) users.^14,26^ Several sensitivity analyses were also performed to test the robustness of our results. First, we compared recent cannabis users to former cannabis users (i.e., history of use but none in the past 30 days) as these groups may be more similar and thus less susceptible to unmeasured confounding. To assess the effect of missing data, both a complete case analysis and a multiple imputation strategy were employed in separate sensitivity analyses. For multiple imputation, predictive mean matching was used, and five data sets were imputed before weighted logistic regression models were constructed and the resultant odds ratios were pooled.^27^

Significance was tested through 2-tailed tests at a significance level of *P* < 0.05 for all described analyses. All data analyses were performed using R v3.5.2 (R Core Team, Vienna, Austria). The sample size was based on the available data, and no a priori power calculations were performed.

## Results

**Figure 1.**
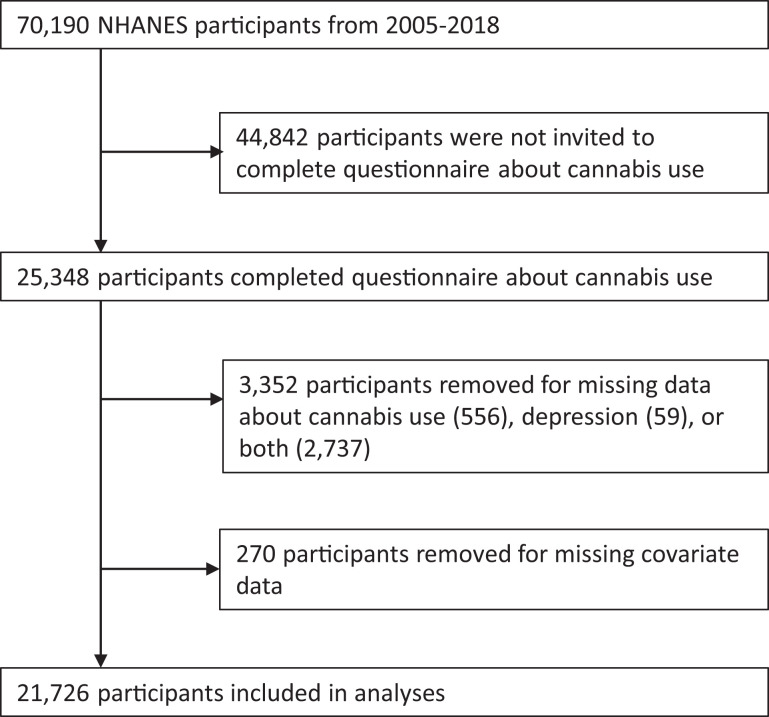
Participant inclusion flowchart (National Health and Nutrition Examination Survey [NHANES]).

Survey data about cannabis use were collected from 25,348 participants. After excluding those with missing outcome, exposure, or covariate data (other than the exceptions described above), 21,726 participants were included in analysis (Figure 1). In the primary analysis, 18,599 (85.6%, weighted) participants reported no use in the past 30 days while 3,127 (14.4%) did. The distribution of cohort characteristics stratified by cannabis exposure is presented in Table 1.

**Table 1.** Characteristics of Participants Included in Study from the National Health and Nutrition Examination Survey (NHANES) 2005 to 2018.

Characteristic	No Use in Past 30 Days (*n* = 18,599)	Used in Past 30 Days (*n* = 3,127)	*P* Value
Age (y)			
20 to 29	4,369 (23.0)	1,312 (41.1)	<0.001
30 to 39	4,678 (23.4)	780 (23.1)	
40 to 49	4,854 (26.5)	578 (19.2)	
50 to 59	4,698 (26.5)	457 (16.6)	
Female sex	9,954 (52.1)	1,192 (37.9)	<0.001
Race			
Hispanic	5,310 (16.8)	493 (11.5)	<0.001
Non-Hispanic white	7,261 (64.3)	1,405 (65.4)	
Non-Hispanic black	3,730 (10.9)	954 (17.0)	
Other	2,298 (8.0)	275 (6.1)	
Education beyond high school	10,787 (64.8)	1,585 (55.4)	<0.001
Marital status			
Never married	4,119 (20.5)	1,274 (37.9)	<0.001
Married or living with partner	11,818 (66.3)	1,363 (47.0)	
Divorced, separated, or widowed	2,662 (13.1)	490 (15.1)	
Number of people in household			
Living alone	1,563 (9.3)	340 (11.5)	<0.001
2 to 4	11,858 (69.2)	2,104 (71.3)	
5+	5,178 (21.5)	683 (17.2)	
Ratio of family income to poverty level			
≤1	3,567 (13.1)	900 (21.4)	<0.001
1 to 3	6,656 (30.8)	1,230 (37.3)	
>3	6,963 (50.1)	799 (35.4)	
Missing data	1,413 (6.0)	198 (5.8)	
Health insurance coverage	13,726 (80.2)	1,968 (37.3)	<0.001
BMI (kg/m^2^)			
<25	5,276 (29.4)	1,244 (40.3)	<0.001
25 to 30	5,927 (31.9)	922 (29.9)	
≥30	7,396 (38.3)	961 (29.1)	
Cigarette use			
Never	11,744 (61.7)	938 (28.9)	<0.001
Former	3,236 (19.6)	525 (20.8)	
Current	3,619 (18.7)	1,664 (50.3)	
Alcohol use			
None	2,375 (11.4)	154 (4.7)	<0.001
Moderate	9,326 (52.2)	1,489 (44.9)	
Heavy	2,230 (12.5)	882 (29.2)	
Missing data	4,668 (24.0)	602 (21.2)	
Cocaine/heroin/meth, at least once	2,697 (16.1)	1,300 (46.1)	<0.001
Diabetes	1,700 (7.9)	171 (4.8)	<0.001
Arthritis	2,933 (16.8)	484 (16.0)	0.345
Cancer	753 (5.1)	118 (5.1)	0.942
Survey year			
2005 to 2006	2,500 (14.3)	324 (12.0)	<0.001
2007 to 2008	2,746 (14.3)	389 (11.3)	
2009 to 2010	2,957 (14.0)	465 (12.4)	
2011 to 2012	2,573 (14.1)	422 (13.1)	
2013 to 2014	2,832 (14.7)	493 (14.3)	
2015 to 2016	2,644 (14.2)	480 (16.8)	
2017 to 2018	2,347 (14.4)	554 (20.0)	

*Note*. All values are displayed as *n* (weighted %). χ^2^ analysis is used to test significance between groups for categorical variables. BMI = body mass index; Meth = methamphetamine.

Of those who had used cannabis in the past 30 days, 5.6% (weighted) self-reported suicidal ideation in the past 2 weeks, compared to 2.9% with no recent use (*P* < 0.001). Similarly, 13.8% of recent users endorsed symptom profiles of moderate-to-severe depression compared to 7.0% of those with no recent use (*P* < 0.001), and 14.9% of recent users had seen a mental health professional in the past 12 months compared to 9.0% with no recent use (*P* < 0.001).

In unadjusted, survey-weighted analysis, recent cannabis users had 1.98 greater odds (95% CI, 1.60 to 2.46, *P* < 0.001) of endorsing suicidal ideation compared to those who had not used in the past 30 days. After adjusting for covariates, recent users had 1.54 greater odds (95% CI, 1.19 to 2.00, *P* = 0.001). Recent users were also more likely to be depressed (adjusted odds ratio [aOR] 1.53, 95% CI, 1.29 to 1.82, *P* = 0.001) and to have seen a mental health professional in the past 12 months (aOR 1.28, 95% CI, 1.04 to 1.59, *P* = 0.023) relative to the comparator group (Table 2).

**Table 2.** Weighted and Adjusted Odds Ratios for Associations between Cannabis Use and Outcomes.

Cannabis Use	Suicidal Ideation in Past 2 Weeks		Depressed (PHQ-9 ≥ 10)		Seen MHP in Past 12 Months	
	OR (95% CI)	*P* Value	OR (95% CI)	*P* Value	OR (95% CI)	*P* Value
Unadjusted						
No use in past 30 days	1 (ref)		1 (ref)		1 (ref)	
Used in past 30 days	**1.98** (1.60 to 2.46)	**<0.001**	**2.11** (1.84 to 2.43)	**<0.001**	**1.76** (1.47 to 2.10)	**<0.001**
Adjusted						
Used in past 30 days	**1.54** (1.19 to 2.00)	**0.001**	**1.53** (1.29 to 1.82)	**<0.001**	**1.28** (1.04 to 1.59)	**0.023**
By sex^a^						
Male	0.99 (0.68 to 1.45)	0.965	1.26 (0.92 to 1.73)	0.153	**1.31** (1.01 to 1.70)	**0.045**
Female	**2.36** (1.69 to 3.30)	**<0.001**	**1.71** (1.34 to 2.18)	**<0.001**	**1.35** (1.04 to 1.74)	**0.022**
By age-group^b^ (years)						
20 to 29	**1.92** (1.24 to 2.96)	**0.004**	**1.74** (1.32 to 2.28)	**<0.001**	1.07 (0.81 to 1.40)	0.646
30 to 39	1.41 (0.88 to 2.27)	0.158	**1.46** (1.08 to 1.98)	**0.014**	1.53 (0.99 to 2.35)	0.053
40 to 49	1.26 (0.77 to 2.06)	0.354	1.39 (0.93 to 2.06)	0.099	1.37 (0.93 to 2.04)	0.113
50 to 59	1.47 (0.80 to 2.70)	0.212	1.44 (0.97 to 2.13)	0.070	1.17 (0.74 to 1.85)	0.491

*Note*. Bolded values are statistically significant at *P* Value < 0.05. MHP = Mental health professional (psychologist, psychiatrist, psychiatric nurse, or clinical social worker); PHQ-9 = Patient Health Questionnaire-9; OR = odds ratio.

^a^ A separate regression model was built for each sex cohort. OR displayed is for recent users, relative to the sex-matched group with no use in the past 30 days.

^b^ A separate regression model was built for each age cohort. OR displayed is for recent users, relative to the age-matched group with no use in the past 30 days.

Subgroup analysis by sex demonstrated that males who used cannabis in the past 30 days were not any more likely to endorse suicidal thinking compared to those without recent use (aOR 0.99, 95% CI, 0.68 to 1.45, *P* = 0.965). In contrast, females with recent use had 2.36 greater odds (95% CI, 1.69 to 3.30, *P* < 0.001) of suicidal thinking compared to females who had no recent cannabis use (Table 2). Another subgroup analysis by age cohort demonstrated that recent cannabis use was associated with greater odds of suicidal thinking in all age groups, though this finding was statistically significant only for the youngest cohort aged 20 to 29 years (aOR 1.92, 95% CI, 1.24 to 2.96, *P* = 0.004; Table 2).

In a secondary analysis, recent users were further characterized as moderate (<20 days in the past month) or heavy users (≥20), and these cohorts respectively had 1.44 (95% CI, 1.10 to 1.90, *P* = 0.009) and 1.74 (95% CI, 1.14 to 2.65, *P* = 0.01) greater odds of experiencing suicidal ideation compared to the cohort with no use in the past 30 days (Table 3). In a sensitivity analysis, recent users remained more likely to have suicidal thoughts (aOR 1.58, 95% CI, 1.16 to 2.17, *P* = 0.004) compared to the subgroup of those who formerly used cannabis but had not in the past 30 days (Table 3).

**Table 3.** Weighted and Adjusted Odds Ratios for Secondary and Sensitivity Analyses between Cannabis Use and Outcomes.

Cannabis Use	Suicidal Ideation in Past 2 Weeks		Depressed (PHQ-9 ≥ 10)		Seen MHP in Past 12 Months	
	OR (95% CI)	*P* Value	OR (95% CI)	*P* Value	OR (95% CI)	*P* Value
Secondary analysis: frequency of use						
No use in past 30 days	1 (ref)				1 (ref)	
Moderate use (<20 days/month)	**1.44** (1.10 to 1.90)	**0.009**	**1.43** (1.18 to 1.74)	**<0.001**	**1.34** (1.08 to 1.65)	**0.007**
Heavy use (≥20 days/month)	**1.74** (1.14 to 2.65)	**0.010**	**1.71** (1.33 to 2.19)	**<0.001**	1.20 (0.86 to 1.67)	0.295
Sensitivity analysis: former use						
Last used >30 days ago	1 (ref)		1 (ref)		1 (ref)	
Used in past 30 days	**1.58** (1.16 to 2.17)	**0.004**	**1.45** (1.20 to 1.75)	**<0.001**	1.24 (1.00 to 1.54)	0.051

*Note*. Bolded values are statistically significant at *P* Value < 0.05. MHP = Mental health professional (psychologist, psychiatrist, psychiatric nurse, or clinical social worker); PHQ-9 = Patient Health Questionnaire-9; OR = odds ratio.

In determining the impact of missing data on the primary results, a complete case analysis retained 15,431 participants, and recent cannabis users had 1.56 greater odds (95% CI, 1.19 to 2.05, *P* = 0.001) of suicidal ideation compared to those with no use in the past 30 days. A subsequent analysis employing multiple imputation of all missing data demonstrated similar effect estimates across five imputations (pooled aOR 1.50, 95% CI, 1.19 to 1.90, *P* < 0.001).

## Discussion

This study demonstrated a significant association between cannabis use in the past 30 days and suicidal ideation in a nationally representative cohort of adults aged 20 to 59. Recent use was also associated with symptom profiles of moderate-to-severe depression and having seen a mental health professional in the past 12 months.

These findings agree with 2 systematic reviews^10,14^ demonstrating that adults with any lifetime cannabis use have greater odds of experiencing suicidal ideation. Borges et al.^14^ further found that heavy cannabis users were more likely to experience suicidal thinking (pooled OR 2.53, 95% CI, 1.00 to 6.39) compared to nonusers. Our own analyses similarly demonstrated that heavy users (at least 20 days per month) were at greater odds of suicidal ideation compared to moderate users and nonusers. However, there was no singular definition of heavy cannabis use across the 5 studies included in the review, ranging widely from 10 lifetime uses to daily use. This makes interpretation and comparison of the observed and pooled effect sizes difficult. There is currently no consensus on a definition of moderate versus heavy cannabis use; further work is needed to define clinically relevant thresholds related to problematic use.

Our sex-stratified results suggest that the above observations may be driven largely by the female cohort. While females generally have lower rates of suicide completion than males, they do have higher incidences of suicidal ideation and attempt.^28^ As cannabis is a strong independent risk factor for the conversion from suicidal ideation to actions,^29^ our finding suggests that timely medical and social intervention may be even more prudent for female users with thoughts of self-harm.

In subgroup analysis by age-group, the association between cannabis use and suicidal ideation was only statistically significant in the youngest group (20 to 29 years). However, the effect sizes in other age groups still suggest a positive association. The relatively low prevalence of cannabis use in older adults may have limited the power of the analysis to demonstrate statistical significance in these groups. Previous work has described a strong association between any lifetime cannabis use and the earlier development of depression and suicidality.^10–12^ However, our cross-sectional data examined suicidality and concurrent use—it is unclear whether cannabis use that started earlier in life contributed to the observed results. This question requires longitudinal investigation with adequately powered data.

This study builds on the findings of a recent study by Gorfinkel et al.^13^ which analyzed similar NHANES data and described 1.90 (95% CI, 1.62 to 2.24) greater odds of depressive symptomatology in respondents who had used cannabis in the past 30 days compared to nonusers. We were able to replicate a similar association between cannabis use and depression, with the difference in effect estimates possibly attributed to our additional inclusion of the 2017 to 2018 survey data and consideration of additional plausible confounding variables in regression modeling such as cigarette smoking, BMI, and select medical comorbidities.^24,25^ Our findings expand on their discussion about the relationship between cannabis and other outcomes related to mental health. We considered suicidality separate from depression as these are overlapping but distinct dimensions of disease.^30^ While we acknowledge that suicidal ideation is not to be equated to attempt or completion, it has been shown to be a strong predictor and considered an appropriate surrogate metric.^31,32^

The relationship between cannabis and mental health outcomes is complex. While observational studies have suggested that heavy cannabis use may unveil depressive or psychotic episodes^12^, there are many biopsychosocial factors involved.^33,34^ This discussion is further complicated by recent trends of cannabis and synthetic cannabinoids being used as self-medication or prescribed as experimental therapy for acute and chronic psychiatric disorders. While there is literature in support of its therapeutic value and safety for chronic pain,^35,36^ multiple sclerosis,^37^ cancer,^38^ and inflammatory bowel disease,^39^ the evidence for prescribing cannabis for the symptomatic treatment of psychotic, anxiety, or mood disorders is scarce and mixed.^40–42^ As our understanding of the physiologic effects of different medical cannabis formulations advances, we should carefully discern the long-term benefits versus harms on psychiatric outcomes in future research.

This study has several limitations inherent in its design that should be considered when interpreting the results. First, the NHANES questions are self-reported; thus, our analyses rely on accurate responses from participants who are willing and able to partake in the survey process. Given historical stigma attached to both cannabis use and mental illness, participants’ responses may have been more biased in the past. However, there was no significant interaction between time and cannabis use, suggesting that changes in this response bias did not influence the result. Second, our regression models were limited by data collected by the NHANES and could not include all relevant outcomes of interest such as suicide attempts and completion. Likewise, not all plausible confounding factors were available for inclusion in regression models, such as chronic pain, psychiatric history, or previous suicidality. Furthermore, we lacked data about cannabis dose and formulations and were unable to distinguish between medicinal and recreational cannabis use, which could introduce unmeasured confounding since cannabis is now being prescribed by some practitioners for the symptomatic treatment of depressive disorders. Finally, large amounts of data missing at random due to nonresponse or missing for systematic reasons (e.g. alcohol questionnaire data not yet published for the 2017 to 2018 cycle) could skew the results. However, we implemented a strategy to retain as much data as possible, and subsequently, both complete case analysis and multiple imputation revealed similar effect sizes suggesting that the missing data did not bias our results.

## Conclusion

Our analyses of a nationally representative sample demonstrate that recent cannabis use is associated with suicidal ideation and moderate-to-severe clinical depression. This relationship is complex and requires further investigation to characterize. To what extent cannabis use leads to suicidality versus severe depression leading to cannabis use remains uncertain. Given the biopsychosocial burden of depression and suicide on individuals and health systems—combined with loosening medicolegal views toward cannabis use—policies and clinical guidelines about cannabis use should be thoughtfully developed and implemented to target improved health outcomes at the patient and population levels. This is especially true for medically prescribed cannabis formulations, and future efforts should work to characterize the long-term benefits and harms of acute and chronic cannabis use, especially for psychiatric therapeutic purposes.
